# Retinal and Corneal Toxicity and Pharmacokinetic Analysis of Intraocular Injection of Ganciclovir in Rabbit Eyes

**DOI:** 10.1155/2019/3054758

**Published:** 2019-05-07

**Authors:** Bin-jia Sun, Rong-mei Peng, Qing Lu, Jing Hong

**Affiliations:** Department of Ophthalmology, Peking University Third Hospital, Beijing 100191, China

## Abstract

**Purpose:**

To evaluate the safety and pharmacokinetic changes of ganciclovir (GCV) intraocular injection.

**Methods:**

GCV (2 mg/0.1 mL) was injected into rabbit eyes. Aqueous GCV concentration was detected by high-performance liquid chromatography. Potential toxicity was assessed by slit-lamp examination, optical coherence tomography, fundus examination, confocal microscopy, and histology.

**Results:**

Aqueous GCV concentrations were 24.83 ± 6.41 *μ*g/mL, 0.65 ± 0.52 *μ*g/mL, and undetected on the 1^st^, 3^rd^, and 7^th^ day after intravitreal injection. GCV could not be detected on the first day after intracameral injection. No corneal abnormality was found after intravitreal injection, but retinal edema was observed on the first day which receded later. Corneal edema was obvious with endothelial cytoarchitecture damaged after intracameral injection; fluid retention also existed in retina.

**Conclusions:**

GCV intravitreal injection offers effective, sustained drug concentration in the anterior chamber, and its damage to retina receded over time. Intracameral injection results in rapid drug elimination and severe damage to endothelium and thus is not recommended.

## 1. Introduction

Intraocular viral infection has become an increasingly serious eye disease, causing visual impairment and even blindness. Viruses can affect any parts of the eyeball and cause various diseases, such as necrotic retinitis, obstinate uveitis, and endotheliitis, resulting in irreversible damage and even blindness. Therefore, a potent antiviral therapy is essential to effectively inhibit virus replication and reduce tissue damage. Although numerous studies have explored treatment of intraocular viral infections, the choice of drugs and administration routes to achieve effective concentrations at the lesion site needs further investigation.

Ganciclovir (GCV), an antiviral medication developed in the 1980s, has a strong antiviral effect against cytomegalovirus (CMV) and other members of the herpes virus family [1]. GCV is a lipophilic drug with a preferential affinity for cell membranes. By inhibiting viral DNA polymerase, GCV terminates the elongation of viral DNA, which in turn arrests viral replication [2].

GCV is always used to treat or prevent infections of CMV and other members of the herpes virus family. Systemic GCV has been widely used in treatment of CMV-related retinitis, and it has also been recognized as an effective treatment for acute inflammation in CMV endotheliitis [3]. However, the concentration of GCV in the eyeball is low because of the blood-ocular barrier [4]. In addition, long-term administration of systemic GCV can lead to a series of systemic complications; the most common serious complication is bone-marrow suppression, which induces severe neutropenia [5, 6].

Topical GCV therapy has been considered as a combination therapy with systemic GCV or as a prophylactic therapy. A topical GCV gel has been approved for treatment of HSV keratitis in more than 30 countries [3]. Topical GCV gel also has proven therapeutic and antiviral effects on CMV endotheliitis [7], with lower costs and fewer side effects. However, the concentration of GCV is also limited in the eyeball because of the corneal barrier.

In the last few years, intravitreal injection of ganciclovir (IVTG) has been widely used in treatment of CMV retinitis. It has also been introduced in treatment of refractory CMV endotheliitis in a case report [8]. This method is relatively easy, has fewer systemic side effects, and provides fast delivery of high intraocular drug concentrations. However, the safety of intraocular administration of GCV for intraocular tissues remains controversial. The aim of this study is to evaluate the safety of intraocular (intravitreal/intracameral) injection of GCV for corneal endothelium and retinal tissue, study the pharmacokinetic changes of GCV after intraocular injection, and provide basis for ganciclovir intraocular injection in the treatment of refractory viral corneal endotheliitis.

## 2. Materials and Methods

A total of 46 New Zealand white rabbits (Beijing Vital River Laboratory Animal Technology Co., Ltd.) were used. 34 rabbits were randomly divided into two groups: one group received intravitreal injection of GCV (2 mg/0.1 mL) and the other group received intracameral injection of GCV (2 mg/0.1 mL). Five of each group were used for anterior chamber GCV concentration detection on the 1^st^, 3^rd^, and 7^th^ day after injection, and the others were used for histological examinations on the 1^st^, 7^th^, 14^th^, and 28^th^ day after injection. The other 12 rabbits were used as control; one eye received intravitreal injection of an equal volume of balanced salt solution (BSS), and the other eye received intracameral injection of BSS. All experiments were performed in accordance with the ethical standards of Peking University Third Hospital's Institutional Review Board and with the Association for Research in Vision and Ophthalmology (ARVO) Resolution on the Use of Animals in Research.

### 2.1. Intraocular Injection

#### 2.1.1. Intravitreal Injection

Rabbits were anesthetized with intramuscular injections of 20 mg/kg ketamine and 5 mg/kg xylazine. After topical applications of 0.4% oxybuprocaine hydrochloride, intravitreal injections were performed. Under sterile conditions, a 27-gauge needle was introduced through the cornea, and 0.1 mL of aqueous humor was removed from the anterior chamber to ensure adequate hypotony. Then, the drug was injected very slowly with a 25-gauge needle 2 mm posterior to the limbus, with care taken to avoid hitting the lens.

#### 2.1.2. Intracameral Injection

Rabbits were anesthetized as previously described. After anterior chamber, paracentesis was performed to maintain normal intraocular pressure and the drug was injected with a 27-gauge needle through the corneal limbus into the anterior chamber.

### 2.2. Examinations

Rabbits in both groups were monitored by slit-lamp examination, optical coherence tomography (IVue-100 Fourier-domain optical coherence tomograph, Optovue Inc., Fremont, CA, USA), microscopy for fundus examination, and confocal microscopy (Heidelberg Retina Tomograph 3 with Rostock Cornea Module; Heidelberg Engineering, Heidelberg, Germany) before and 1, 7, 14, and 28 days after intraocular injection of GCV to detect changes (corneal edema, intraocular inflammation, vitreous haze, vitreous hemorrhage, retinal detachment, preretinal membrane formation, or neovascularization).

### 2.3. Detection of Ganciclovir Concentration in Aqueous Humor

A pharmacokinetic study was carried out to determine changes in GCV concentration and how long it remained in the aqueous humor. Before aqueous humor collection, animals were anesthetized as previously described. Anterior chamber paracentesis was performed with a 27-gauge needle introduced through the corneal limbus, and 0.1 mL of aqueous humor was removed from the anterior chamber. The samples were frozen and stored at −80°C until analyzed.

The GCV concentration of rabbit aqueous humor was detected by a high-performance liquid chromatography (HPLC) assay with electrochemical detection. Samples were centrifuged, and the supernatant was injected into a hypersil silica column (100 × 4.6 mm) after dilution. Then, we recorded the peak area and calculated the concentration of GCV. The limit of sensitivity of the assay was 1 *μ*g/mL.

### 2.4. Histological Studies

Rabbits were euthanized at the scheduled time by an intravenous overdose of sodium pentobarbital, and their eyeballs were immediately extracted. Corneal and retinal samples were fixed in fixative.

#### 2.4.1. HE Staining

A portion of corneal and retinal tissue was fixed in 4% formaldehyde solution for 24 h at room temperature. Specimens were dehydrated in a series of ascending concentrations of ethanol, cleared in xylene, and embedded in paraffin wax. Serial sections of the eye were cut at a thickness of 4.0 *μ*m and mounted on glass slides. After being dewaxed in xylene, sections were hydrated in a series of descending concentrations of ethanol. The hydrated sections were stained with hematoxylin solution for 10 minutes, rinsed in tap water for 15 minutes, immersed in 0.5% eosin solution for 10 minutes, dehydrated in a series of ascending concentrations of ethanol, cleared in xylene, and mounted in synthetic resin solution [9]. All sections were examined in detail by light microscopy (Nikon, Tokyo, Japan).

#### 2.4.2. Scanning Electron Microscopy

A portion of corneal and retinal tissue was fixed in 3% glutaraldehyde in PBS for 2 h at room temperature, washed three times with PBS, and then dehydrated through a graded series of ethanol. Samples were critical-point dried, sputter coated with 20 nm gold-palladium, and examined with scanning electron microscopy (SEM; JSM-5600LV; JEOL, Tokyo, Japan).

#### 2.4.3. Transmission Electron Microscopy

Samples were fixed with 3% glutaraldehyde for 1 h at room temperature, fixed with fresh 3% glutaraldehyde once, postfixed with 1% osmium tetroxide, washed three times in PBS for 5 minutes each, dehydrated through a graded series of ethanol, and embedded in Epon 812. Ultrathin (80 nm) sections were collected on copper grids and double-stained with uranyl acetate and lead citrate. Then, sections were examined with transmission electron microscopy (TEM; JEM-1230; JEOL).

### 2.5. Statistical Analysis

The data were analyzed with SPSS Statistics 20. Aqueous GCV concentrations and endothelial cell density (ECD) were analyzed separately. To compare the ECD of the cornea at different time points between all these groups, ANOVA was employed. Data are presented as mean ± standard deviation. *P* values of less than 0.05 were considered as statistically significant.

## 3. Results

### 3.1. GCV Concentration in Aqueous Humor

**Figure 1:**
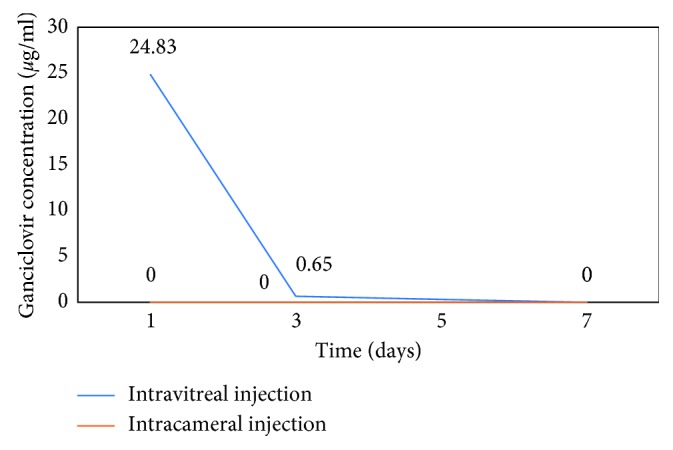
Ganciclovir concentration in aqueous humor was detected on the 1^st^, 3^rd^, and 7^th^ day after intravitreal/intracameral injection of 2 mg/0.1 mL GCV, separately.

Aqueous GCV concentrations were 24.83 ± 6.41 (16.51∼34.02) *μ*g/mL on the first day after intravitreal injection and 0.65 ± 0.52 (0∼1.18) *μ*g/mL on the third day after injection. GCV could not be detected in any samples on the 7^th^ day after injection. Unexpectedly, GCV could not be detected in the aqueous even on the first day after intracameral injection (Figure 1).

### 3.2. Slit-Lamp Examination, Optical Coherence Tomography, and Fundus Examination

**Figure 2:**
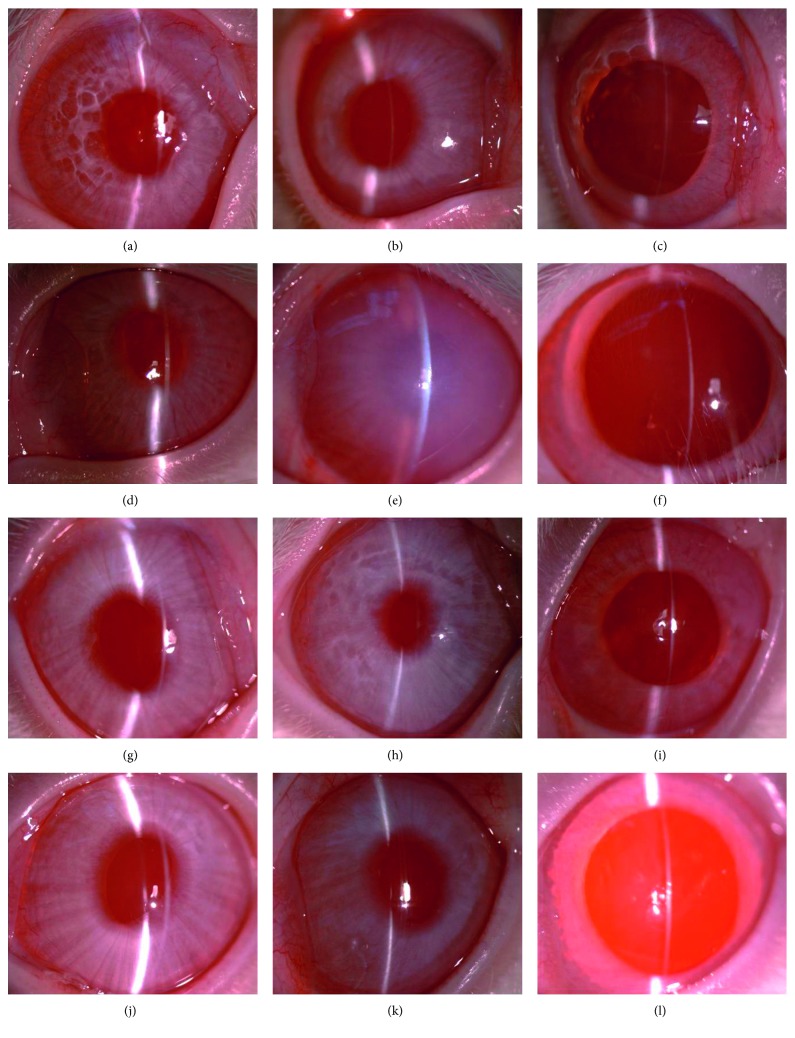
Representative anterior-segment photographs of rabbit eyes. Photographs of a rabbit eye (a) before injection and (b) 1 day and (c) 7 days after intravitreal injection of 2 mg/0.1 mL GCV. Photographs of a rabbit eye (d) before injection and (e) 1 day and (f) 7 days after intracameral injection of 2 mg/0.1 mL GCV. Photographs of a rabbit eye (g) before injection and (h) 1 day and (i) 7 days after intravitreal injection of 0.1 mL BSS. Photographs of a rabbit eye (j) before injection and (k) 1 day and (l) 7 days after intracameral injection of 0.1 mL BSS. Original magnification, ×16.

**Figure 3:**
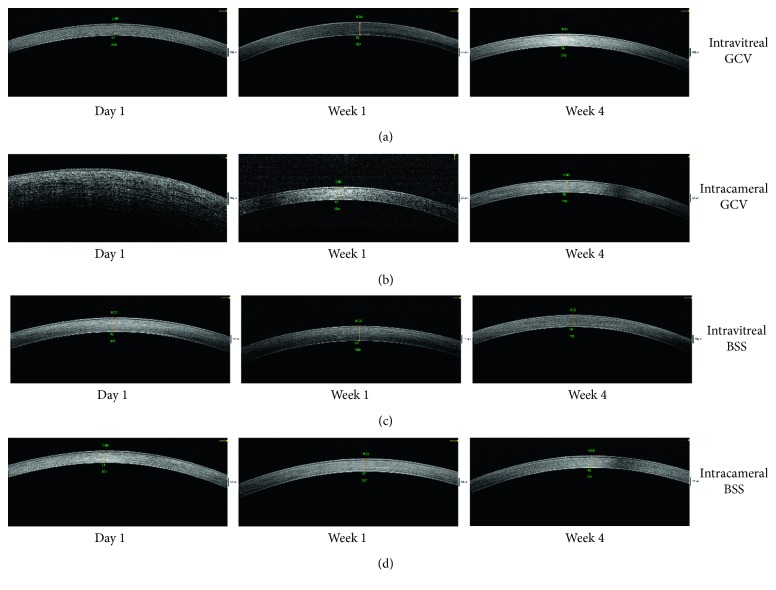
Representative optical coherence tomography of rabbit eyes. (a) Optical coherence tomography of a rabbit eye 1 day, 1 week, and 4 weeks after intravitreal injection of 2 mg/0.1 mL GCV. (b) Optical coherence tomography of a rabbit eye 1 day, 1 week, and 4 weeks after intracameral injection of 2 mg/0.1 mL GCV. (c) Optical coherence tomography of a rabbit eye 1 day, 1 week, and 4 weeks after intravitreal injection of 0.1 mL BSS. (d) Optical coherence tomography of a rabbit eye 1 day, 1 week, and 4 weeks after intracameral injection of 0.1 mL BSS.

Slit-lamp examination, optical coherence tomography, and microscopy for fundus examination of the rabbit eyes after intravitreal injection of 2 mg/0.1 mL GCV showed no corneal edema, intraocular inflammation, or any other abnormalities (Figures 2 and 3). However, slit-lamp examination showed diffuse corneal edema on the first day after intracameral injection of GCV. The cornea recovered clarity within one week (Figure 2). Optical coherence tomography also showed that the cornea significantly thickened on the first day after injection and got normal within one week (Figure 3). No abnormality was found in the rabbit eyes after intravitreal/intracameral injection of 0.1 mL BSS (Figures 2 and 3). Microscopy for fundus examination did not reveal any abnormalities in all rabbit eyes (results not shown).

### 3.3. Confocal Microscopy

**Figure 4:**
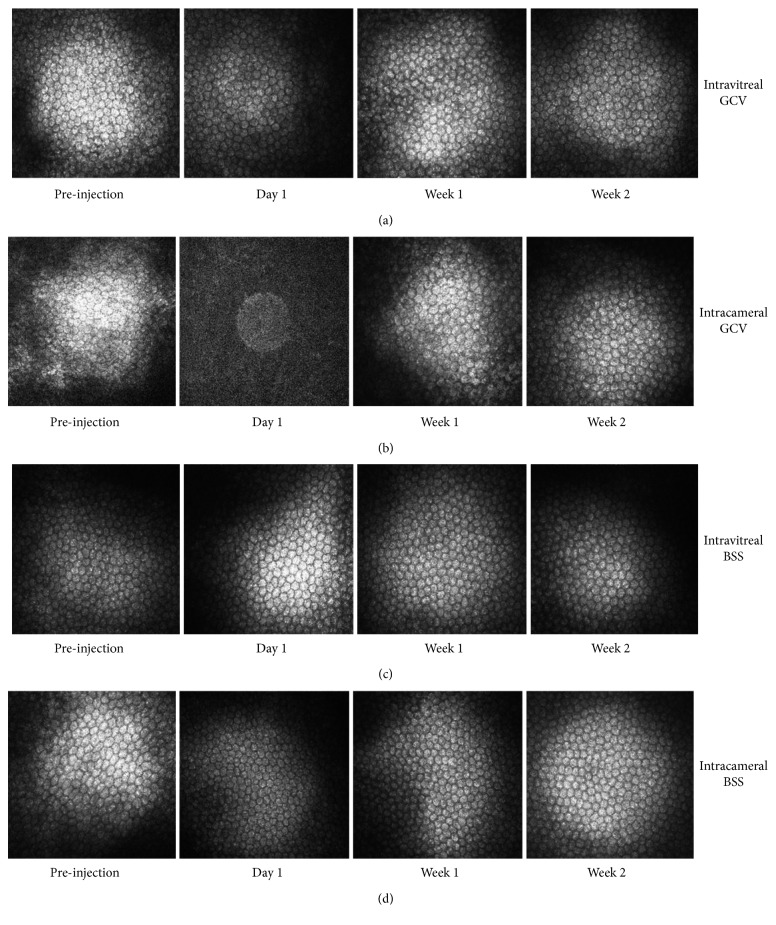
Representative confocal microscopy of the endothelial layers of rabbit eyes. (a) Confocal microscopy of a rabbit eye before and after intravitreal injection of 2 mg/0.1 mL GCV. (b) Confocal microscopy of a rabbit eye before and after intracameral injection of 2 mg/0.1 mL GCV. (c) Confocal microscopy of a rabbit eye before and after intravitreal injection of 0.1 mL BSS. (d) Confocal microscopy of a rabbit eye before and after intracameral injection of 0.1 mL BSS.

Endothelial cells were typically hexagonal before injection and 1 day, 1 week, and 2 weeks after intravitreal injection of 2 mg/0.1 mL GCV (Figure 4). The mean ECD of the intravitreal injection group was 2765 ± 358 cells/mm^2^ before injection. On the first, 7^th^, and 14^th^ day after injection, the mean ECD was 2791 ± 295 cells/mm^2^, 2702 ± 160 cells/mm^2^, and 2788 ± 283 cells/mm^2^, respectively.

We were unable to quantify the ECD of three of all rabbits on the first day after intracameral injection of GCV due to corneal edema (Figure 4). The cornea recovered its clarity, and hexagonal cells were detected on the 7^th^ day after injection. The ECD was 2864 ± 401 cells/mm^2^, 3029 ± 366 cells/mm^2^, 2828 ± 378 cells/mm^2^, and 2575 ± 74 cells/mm^2^ before injection and 1 day, 1 week, and 2 weeks after injection, respectively.

In the control group, endothelial cells were also typically hexagonal before and after intraocular injection of an equal volume of BSS (Figure 4). The mean ECD was 2603 ± 180 cells/mm^2^, 2557 ± 96 cells/mm^2^, 2766 ± 171 cells/mm^2^, and 3013 ± 482 cells/mm^2^ before and 1 day, 1 week, and 2 weeks after intravitreal injection of BSS. The mean ECD was 2812 ± 251 cells/mm^2^, 3097 ± 244 cells/mm^2^, 2802 ± 356 cells/mm^2^, and 2586 ± 267 cells/mm^2^ before and 1 day, 1 week, and 2 weeks after intracameral injection of BSS.

There was no significant difference in ECD before injection (*P*=0.681, *F* = 0.510) and 1 day (*P*=0.052, *F* = 3.372), 1 week (*P*=0.894, *F* = 0.200), and 2 weeks (*P*=0.237, *F* = 1.619) after injection between these groups.

### 3.4. Light Microscopy

**Figure 5:**
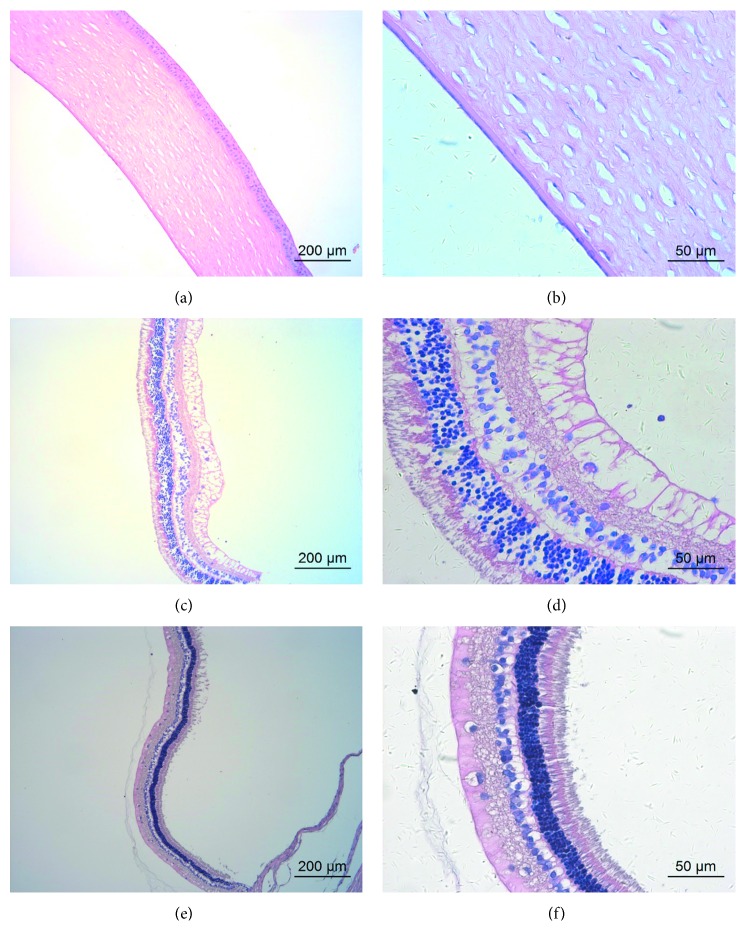
Representative light microscopy of the cornea and retina of a rabbit eye after intravitreal injection of 2 mg/0.1 mL GCV. (a, b) Corneal morphology 1 day after intravitreal injection at different magnifications. (c, d) Retinal morphology 1 day after intravitreal injection at different magnifications. (e, f) Retinal morphology 2 weeks after intravitreal injection at different magnifications. Hematoxylin and eosin stain.

Light microscopy revealed normal corneal morphology after intravitreal injection of GCV (Figures 5(a) and 5(b)). However, there were some edematous changes in retinal tissues. The nerve fiber layer was edematous and thickened. Moreover, the nuclear layers and photoreceptor layer were loosely arranged (Figures 5(c) and 5(d)). The structure of the retina returned to normal 2 weeks after injection (Figures 5(e) and 5(f)).

**Figure 6:**
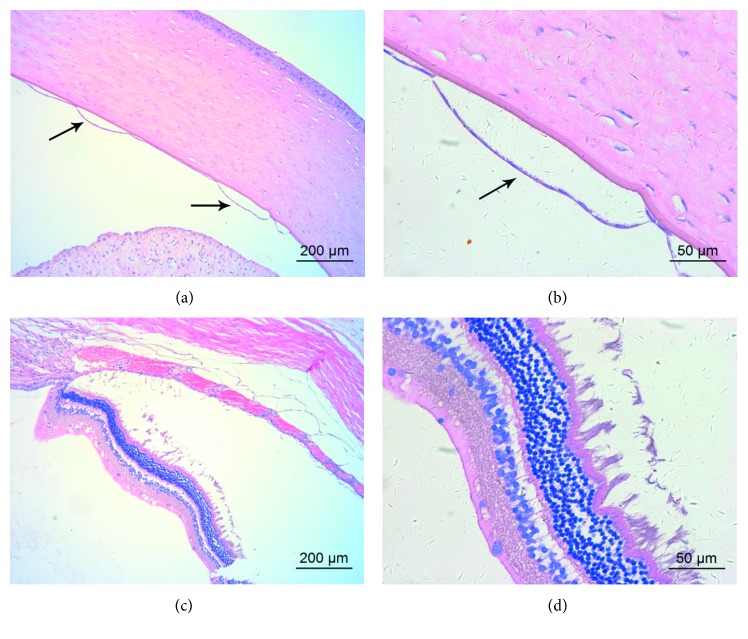
Representative light microscopy of the cornea and retina of a rabbit eye 1 day after intracameral injection of 2 mg/0.1 mL GCV. (a, b) Corneal morphology at different magnifications. The endothelium was separated from Descemet's membrane (black arrows). (c, d) Retinal morphology at different magnifications.

After intracameral injection of GCV, the corneal stroma layer was obviously thickened. The endothelium was separated from Descemet's membrane during tissue processing (Figures 6(a) and 6(b)). The morphology of retinal tissue was normal. The photoreceptor layer was intact, and the nuclear layers remained well organized (Figures 6(c) and 6(d)).

**Figure 7:**
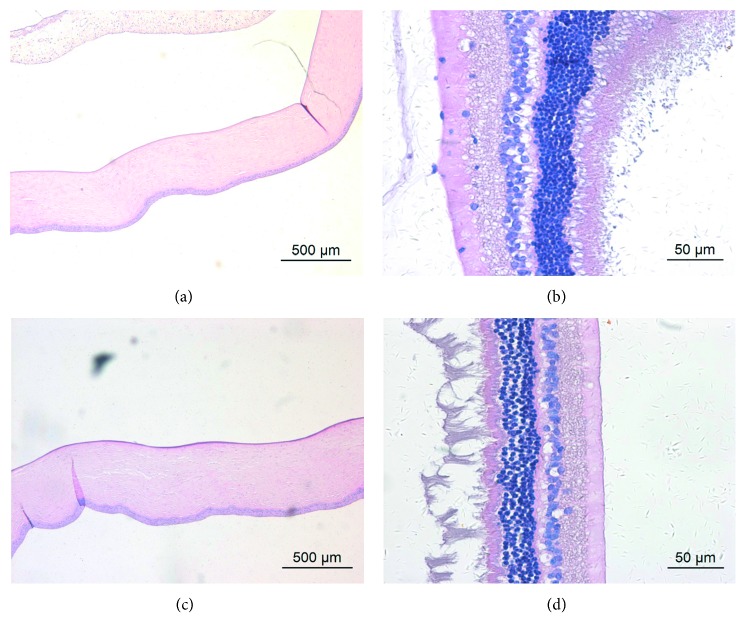
Representative light microscopy of the cornea and retina of rabbit eyes 1 day after intraocular injection of 0.1 mL BSS at different magnifications. (a, b) Corneal and retinal morphology after intravitreal injection. (c, d) Corneal and retinal morphology after intracameral injection.

The morphology of corneal and retinal tissue was normal after intravitreal/intracameral injection of BSS (Figure 7).

### 3.5. Scanning Electron Microscopy

**Figure 8:**
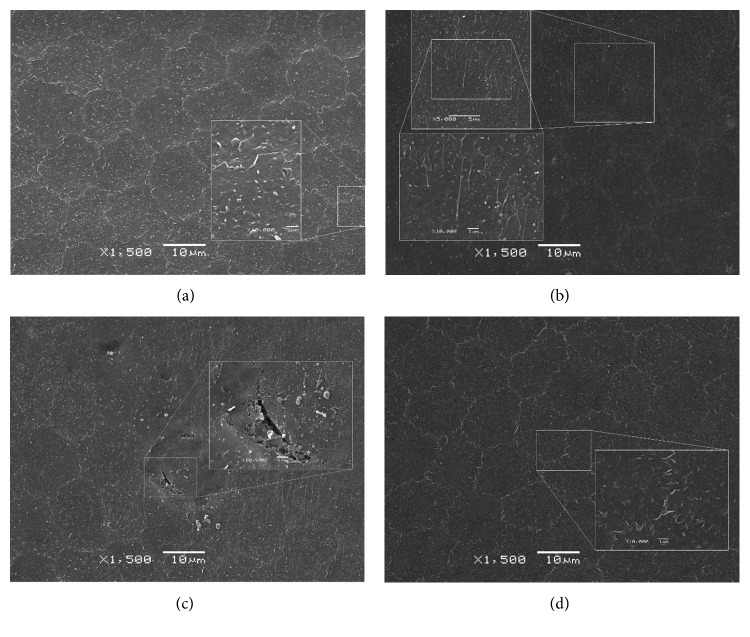
Representative scanning electron microscopy of the endothelial layers of rabbit eyes after intravitreal injection of GCV (a), intracameral injection of GCV (b, c), and intracameral injection of BSS (d).

SEM showed no corneal endothelial abnormalities after intravitreal injection of GCV. Corneal endothelial cells exhibited normal cytoarchitecture. The boundary of hexagonal cells was clear, and intercellular connections were tight. Endothelial cells intersected with each other. Microvilli on the cell surface appeared normal (Figure 8(a)).

In contrast, the boundary of corneal endothelial cells was unclear after intracameral injection of GCV. Intercellular connections were destroyed, and microvilli on the cell surface were shortened and abnormal (Figure 8(b)). Some cells even disintegrated and disappeared, leaving the elastic layer exposed (Figure 8(c)).

Corneal endothelial cells exhibited normal cytoarchitecture after intravitreal injection of BSS (results not shown) and intraocular injection of BSS (Figure 8(d)).

### 3.6. Transmission Electron Microscopy

**Figure 9:**
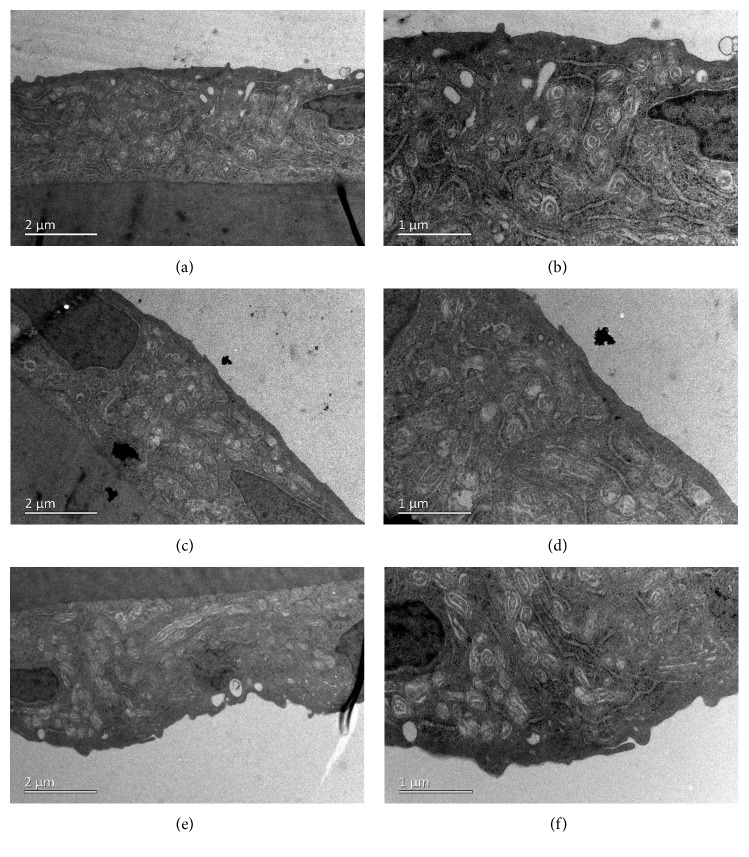
Transmission electron microscopy of the corneal endothelial layer of a rabbit eye at different magnifications on the 1^st^ day (a, b), 7^th^ day (c, d), and 28^th^ day (e, f) after intravitreal injection of 2 mg/0.1 mL GCV.

**Figure 10:**
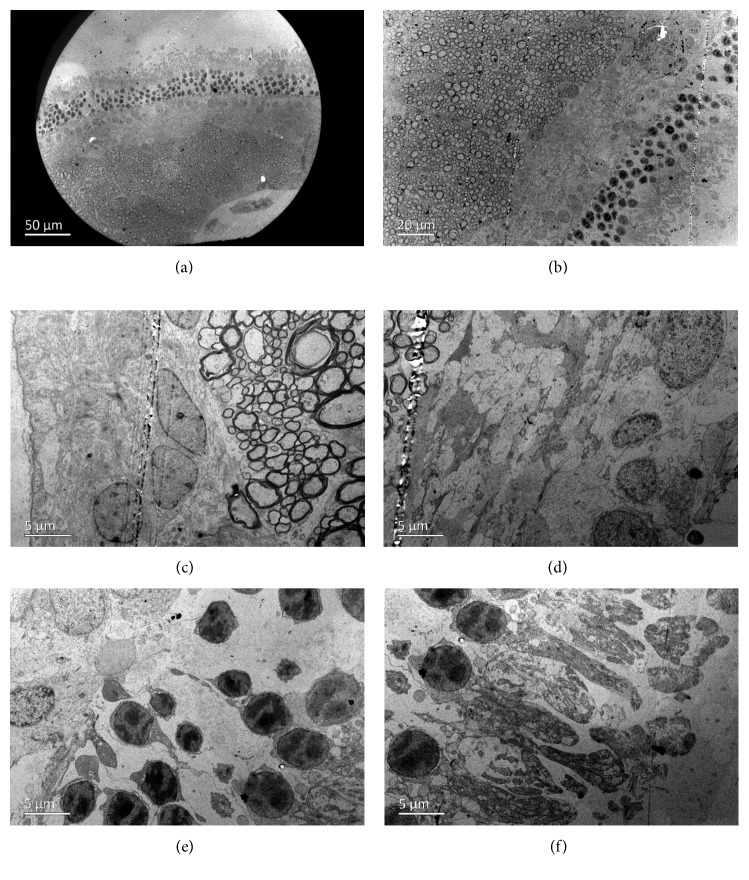
Transmission electron microscopy of the retina of a rabbit eye on the first day after intravitreal injection of 2 mg/0.1 mL GCV. (a, b) All retinal layers exhibited significant edema at different magnifications. (c) Bubble-like structures were found in the retinal nerve fiber layer. (d, e) The intercellular space was dilated in the inner/outer nuclear layer. (f) Photoreceptors were swollen and loosely arranged, and photoreceptor outer segments were decreased in number.

**Figure 11:**
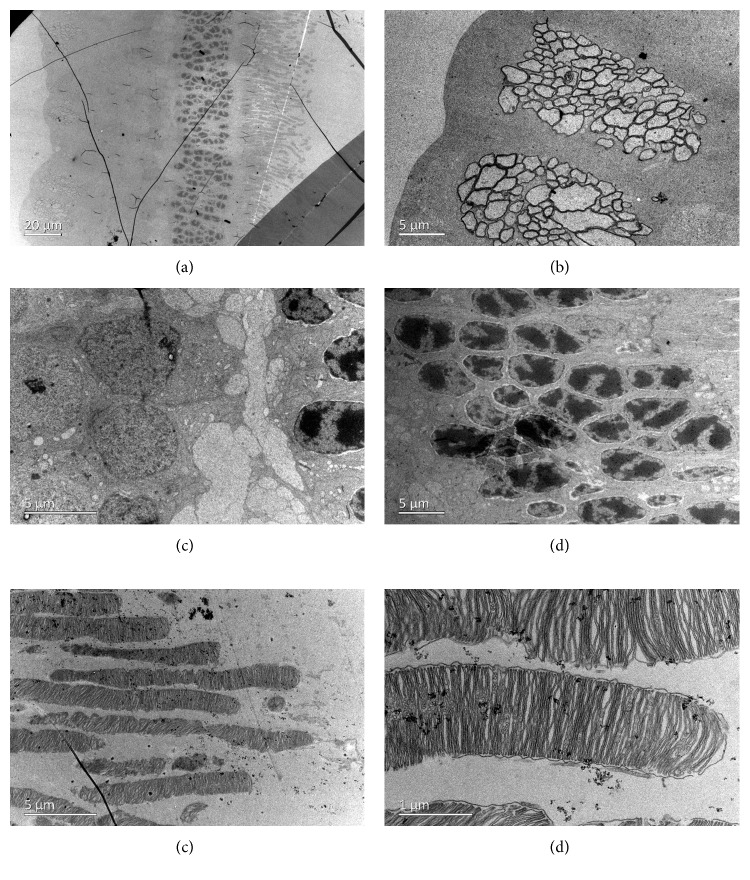
Transmission electron microscopy of the retina of a rabbit eye on the 7^th^ day after intravitreal injection of 2 mg/0.1 mL GCV. (a) Retinal edema remitted on the 7^th^ day after injection. (b) Bubble-like structures remained in the retinal nerve fiber layer but were significantly reduced. (c, d) The intercellular space was smaller in the inner/outer nuclear layers. (e, f) The photoreceptors were less swollen at different magnifications.

**Figure 12:**
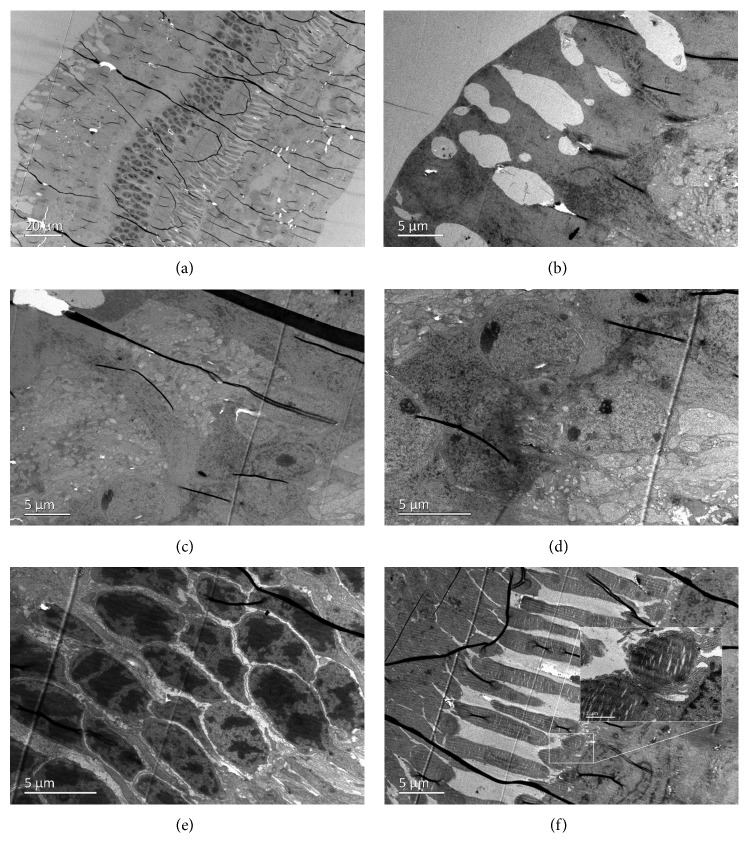
Transmission electron microscopy of the retina of a rabbit eye 4 weeks after intravitreal injection of 2 mg/0.1 mL GCV. (a) Retinal architecture was almost normal except for remaining fluid retention in the retinal nerve fiber layer (b). The inner plexiform layer (c), inner nuclear layer (d), outer nuclear layer (e), and photoreceptor layer (f) all exhibited normal architecture.

TEM showed normal architecture of all the corneal layers after intravitreal injection of 2 mg/0.1 mL GCV. The corneal endothelial cells exhibited normal cytoarchitecture with tight intercellular junctions (Figure 9). However, the retinal tissues exhibited significant edema on the first day after intravitreal injection. The intercellular space was dilated in all layers of the retina. Bubble-like structures were found in the retinal nerve fiber layer. Photoreceptors were swollen and loosely arranged, and photoreceptor outer segments were decreased in number (Figure 10). Retinal tissues and cytoarchitecture were still swollen 1 week after injection (Figure 11). Fortunately, retinal edema gradually receded over time. However, fluid retention remained in the retinal nerve fiber layer 4 weeks after injection (Figure 12).

**Figure 13:**
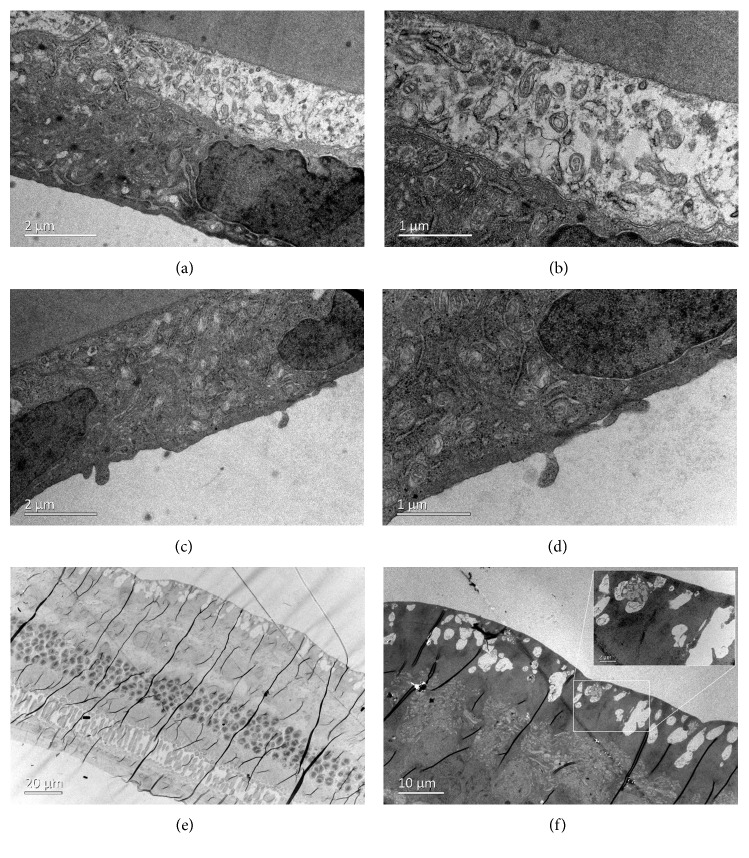
Transmission electron microscopy of the corneal endothelium and retina of a rabbit eye after intracameral injection of 2 mg/0.1 mL GCV. The cytoarchitecture of corneal endothelial cells on the 1^st^ day (a, b) and 7^th^ day (c, d) after injection at different magnifications. (e, f) Fluid retention was still observed in the retinal nerve fiber layer 4 weeks after intracameral injection of 2 mg/0.1 mL GCV.

Endothelial cells were swollen and even broken on the first day after intracameral injection (Figure 13(a)). The mitochondria, golgi body, and endoplasmic reticulum were all swollen (Figure 13(b)). The corneal endothelial cells exhibited normal cytoarchitecture on the 7^th^ day after injection (Figures 13(c) and 13(d)). TEM of retinal tissue revealed fluid retention in the retinal nerve fiber layer (Figures 13(e) and 13(f)), implying that intracameral GCV injection also influenced the retina.

**Figure 14:**
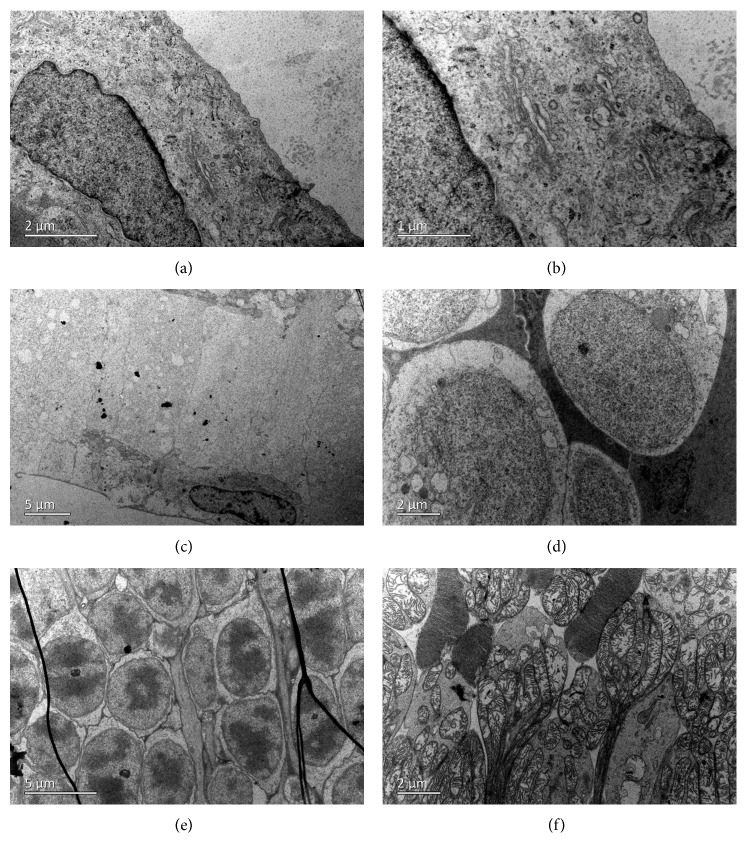
Transmission electron microscopy of the corneal endothelium and retina of rabbit eyes after intraocular injection of 0.1 mL BSS. (a, b) The cytoarchitecture of corneal endothelial cells on the 1^st^ day after intracameral injection. The retinal nerve fiber layer (c), inner nuclear layer (d), outer nuclear layer (e), and photoreceptor layer (f) all exhibited normal architecture after intravitreal injection.

TEM also showed that the corneal and retinal tissue of rabbit eyes exhibited normal cytoarchitecture after intravitreal/intracameral injection of 0.1 mL BSS (Figure 14).

## 4. Discussion

In the last few years, intravitreal injection of GCV has been widely used in treatment of CMV retinitis. Intravitreal injection of GCV has also been introduced in treatment of corneal endotheliitis. This method involves injection of GCV directly into the vitreous body, bypassing all the external barriers. Despite its invasive nature and other complications associated with intravitreal injections, this method of administration remains expedient, provides fast delivery of high intraocular drug concentrations, and has fewer systemic side effects.

### 4.1. Pharmacokinetic Study of GCV

In our study, GCV concentrations in the aqueous humor of rabbits after intravitreal injection were evaluated. Aqueous GCV concentrations were 16.51∼34.02 (24.83 ± 6.41) *μ*g/mL on the first day after injection, approximately 200 times higher than the effective antiviral concentration of GCV (80 ng/mL) [3], and 0∼1.18 (0.65 ± 0.52) μg/mL on the third day after injection, which is also much higher than 80 ng/mL. The drug could not be detected on the 7^th^ day after injection; however, as the limit of sensitivity of the assay was 1 *μ*g/mL, the possibility that some drug remained could not be excluded. Drugs injected into the vitreous diffuse across the vitreous to enter the posterior chamber and anterior chamber. When the drug is injected into the vitreous, a depot is formed and releases the drug in a relatively sustained manner [10], resulting in a steep increase in the apparent elimination half-life.

Schulman also studied the clearance of GCV in aqueous humor following intravitreal injection of 400 *μ*g/0.2 mL in a rabbit model; the GCV concentration was still greater than the in vitro ID50 for several strains of human CMV even 60 hours after injection [11], which indicated the high concentration and prolonged elimination half-time of GCV after intravitreal injection. This phenomenon was in accordance with our results. Peyman determined the intravitreal clearance of liposome-encapsulated GCV. The residual amount of GCV present in the vitreous cavity after a single intravitreal injection of 84.1 *μ*g/0.1 mL was 45.68 ± 5.61 *μ*g/mL, 28.36 ± 3.74 *μ*g/mL, 19.52 ± 2.84 *μ*g/mL, and 8.37 ± 0.20 *μ*g/mL on the first day, 7^th^ day, 14^th^ day, and 28^th^ day after injection, respectively, which was greater than the ID50 for many strains of CMV and VZV [12]. This finding also demonstrated the prolonged half-life of GCV.

The concentration of GCV is significantly lower after topical administration on the ocular surface than after intravitreal injection. The peak concentrations of GCV in the aqueous humor after administration of 0.2% GCV in situ gelling eye drops and common GCV eye drops were 4.79 *µ*g/mL and 0.96 *µ*g/mL, respectively, and the half-lives of GCV in the aqueous humor were 59 minutes and 43 minutes, respectively [13]. A single drop of 0.15% GCV ophthalmic gel, instilled on the ocular surface, can produce a maximal tissue concentration in aqueous humor as high as 1 *µ*g/g [14]. The majority of the administered drug is cleared rapidly from the ocular surface [15]. Several factors contribute to the low bioavailability of drugs administered by this route, including tear fluid turnover and blinking, selective permeability of the corneal epithelial barrier, and drug loss through nasolacrimal and systemic circulation [10].

In our study, we also evaluated GCV concentrations in the aqueous humor of rabbits after intracameral injection. Notably, GCV could not be detected on the first day after intracameral injection, which suggests that GCV cannot remain in the anterior chamber for a long time. Gunda et al. once placed a well on the cornea of rabbits with linear probes implanted in the aqueous humor and allowed 200 *μ*L 0.43% GCV solution to diffuse for 2 h. The GCV concentration in the aqueous humor was just 34 ± 10 nmol/mL (8.7 ± 2.6 *µ*g/mL) 410 minutes later [16]. Elimination of drugs from the aqueous humor was rapid. This rapid elimination occurred because of the turnover of GCV through the chamber angle and Schlemm's canal and by the venous blood flow of the anterior uvea. The turnover rate of aqueous humor in the rabbit eye is 1.5% of the anterior chamber volume per minute, which translates to a half-life of 46 minutes [10], causing the rapid elimination of GCV. Thus, intracameral injection of GCV may be not a good choice for therapy.

### 4.2. Toxicity Evaluation

Currently, intravitreal injection of GCV is widely used in the treatment of CMV retinitis. When taking the severity of retinal lesions in CMV retinitis into account, intravitreal injection of GCV constitutes a safe and efficient treatment for this disease. Recently, GCV was used to treat endotheliitis in a case report. However, whether intravitreal administration of GCV is safe for intraocular tissues remains controversial.

Several clinical studies have evaluated the safety of retina after intravitreal injection of GCV. The reported dose varies from 0.2 to 5 mg of GCV [6, 17–22]. In 2005, Yutthitham reported high-dose (4 mg/0.1 mL), alternating-week intravitreal injections of GCV for treatment of CMV retinitis, and no significant retinal toxicity was found [6]. However, Choopong reported a case of crystallization of 4 mg intravitreal GCV injection leading to retinal arterial occlusion and optic atrophy in 2010, indicating that 4 mg GCV could result in toxicity [23]. Intravitreal injection of 2 mg GCV has been reported several times and has not demonstrated any significant effect on visual acuity. With consideration of the most common dosage used in clinical practice and toxicity from high-dose GCV, we consequently chose a dosage of 2 mg for intraocular injection.

In our study, the retina showed significant tissue edema and swollen cytoarchitecture 1 day after intravitreal injection of GCV. Fortunately, retinal edema gradually receded over time. However, TEM showed that fluid retention remained in the retinal nerve fiber layer 4 weeks after injection. The results indicated that intravitreal injection of 2 mg GCV had a toxic effect on retina; however, the damage to the retina receded over time and may be reversible. Fortunately, there were no abnormal changes in the cornea. As the damage caused by GCV receded over time, it might explain why there was no significant effect on visual acuity in clinical practice in patients with CMV retinitis. This finding also suggested that intravitreal injection of GCV should be used with great caution for treatment of endotheliitis.

Pulido investigated the retina toxicity of GCV intravitreal injection in the rabbit. Intravitreal doses of 400 *μ*g produced no discernible ophthalmoscopic or histologic changes and no changes in electroretinography B-waves [24]. Peyman also demonstrated no evidence of retinal toxicity by clinical or light microscopic examination at different time intervals up to 14 days after intravitreal injections of liposome-encapsulated GCV (94 *µ*g/0.1 mL) and trifluridine (102 *µ*g/0.1 mL) [25]. As their dosage was much lower than ours, no abnormal changes after intravitreal injection were reasonable.

Nevertheless, Moschos revealed that intravitreal injection of GCV doses of 300–600 *μ*g/0.1 mL had a clearly toxic effect on the retina. The electroretinogram was either extinguished or clearly affected one month after injection. Electron microscopy revealed degenerative lesions in all retinal layers. These changes were more pronounced in the outer nuclear layer and less in the remainder of the retina. Notably, GCV shows a clearly toxic effect on the retina even at a dose of 200 *μ*g/0.1 mL 4 months after its delivery [26]. Such findings disagree with other studies, but we should pay attention to the safety of intravitreal administration of GCV.

In our study, we found corneal edema on the first day after intracameral injection of GCV, and we were unable to quantify the ECD of rabbits due to corneal edema. The cytoarchitecture of endothelial cells was nearly destroyed. The cornea recovered clarity within one week because the corneal endothelial cell of rabbits could regenerate with the support of Descemet's membrane [27]. However, human endothelial cells can barely regenerate. A cytotoxicity test showed that GCV had a dose-dependent cytotoxic effect on human corneal endothelial cells. Intracameral GCV concentrations of ≥5 mg/mL may increase the risk of corneal endothelial cell damage, while GCV concentrations of ≤0.5 mg/mL do not decrease cell viability [28]. Intracameral injection of GCV may result in irreversible endothelial dysfunction. In addition, TEM revealed fluid retention in the retinal nerve fiber layer. Intracameral administration of 2 mg GCV had a toxic effect on both the cornea and retina; thus, intracameral administration of GCV is not recommended.

## 5. Conclusion

In conclusion, intravitreal injection of GCV is optional for treatment of refractory viral corneal endotheliitis. This method offers effective drug concentrations in the anterior chamber and forms a drug depot that releases the drug in a sustained manner. Notably, intravitreal injection of GCV has a toxic effect on the retina. Although the damage to the retina receded over time, intravitreal injection should be used with great caution for treatment of endotheliitis. However, intracameral injection of GCV results in rapid elimination of drugs from the aqueous humor and can severely damage endothelial cells; moreover, GCV can induce endothelial dysfunction in humans. Therefore, intracameral injection of GCV is not recommended.

## Data Availability

The data used to support the findings of this study are included within the article.
